# SNP haplotyping technique for evaluation of MGP 5′ UTR power in osteoblast cells

**DOI:** 10.1186/s40064-016-2329-8

**Published:** 2016-06-13

**Authors:** Abazar Roustazadeh, Seyed Hamidreza Monavari, Seyed Reza Hosseini Fard, Parisa Hassanpour, Amaneh Yarnazari, Mohammad Najafi

**Affiliations:** Research Center for Non-Communicable Diseases and Biochemistry Department, Jahrom University of Medical Sciences, Jahrom, Iran; Cellular and Molecular Research Center, Iran University of Medical Sciences, Tehran, Iran; Virology Department, Iran University of Medical Sciences, Tehran, Iran; Biochemistry Department, Iran University of Medical Sciences, Tehran, Iran

**Keywords:** Haplotype, Matrix Gla protein (MGP), Single nucleotide polymorphism (SNP), Osteoblast, Promoter

## Abstract

Matrix Gla protein (MGP) is involved in calcium trafficking and arterial calcification. The aim of study was to investigate the role of three polymorphisms within the MGP gene promoter region on reporter gene (luciferase) expression level. The fragments containing rs1800799 (C/T), rs1800802 (T/C), and rs1800801 (G/A) sites were constructed and transferred into human G292 osteoblast cells using pGL3-Basic plasmid. The reporter gene expression was calculated for the high and low frequency polymorphic haplotypes (CTG and TCA, respectively). Results showed that the reporter gene expression levels are not statistically different (p > 0.3). We concluded that the investigated polymorphic sites are not able to change the gene expression pattern in human G292 osteoblast cells.

## Background

Vascular mineralization is a passive end-stage process in stenosis of arteries (Schurgers et al. 2013). Vitamin K-dependent matrix Gla protein (MGP) is known a calcium scavenger and may play a dominant role in vascular calcium metabolism. It is a mineral-binding extracellular matrix (ECM) protein mostly expressed by vascular smooth muscle cells (VSMCs), osteoblasts and chondrocytes. It is generally believed as potent inhibitor of arterial calcification (Schurgers et al. 2008). A study showed that MGP knockout (MGP^−/−^) mice developed blood vessel rupture and died due to arterial calcification after 2 months (Luo et al. 1997). The MGP gene (Entrez; Gene ID: 4256) is located on the short arm of chromosome 12 (12p12.3). dbSNP data (www.ncbi.nlm.nih.gov/snp) showed that polymorphic sites are widely distributed in the different regions of MGP gene and are potentially related to the MGP function (Farzaneh-Far et al. 2001). Furthermore, the MGP rs1800801 and rs1800802 promoter–reporter gene constructs showed that the rs1800802 *C* allele consistently reduces promoter activity in vascular smooth muscle cells (Herrmann et al. 2000).

Although haplotype blocks are referred as variant distributions in several loci but we defined the haplotypes based on the distribution of polymorphisms within transcription factor elements located separately on the MGP promoter. In previous study, we predicted the high minimal allele frequency (MAF) polymorphic scores for each transcription factor element and showed that the rs1800801, rs1800802 and rs1800799 haplotypes within the MGP promoter did not relate to stenosis of coronary arteries. Moreover, serum MGP concentration was not associated to the genotype and haplotype distributions (Najafi et al. 2014). In this study, we followed our investigations and focused on the promoter expression power using polymorphic constructs in human MGP high expressed G292 osteoblast cells.

## Methods

### High and low frequent haplotype constructs

**Fig. 1:**
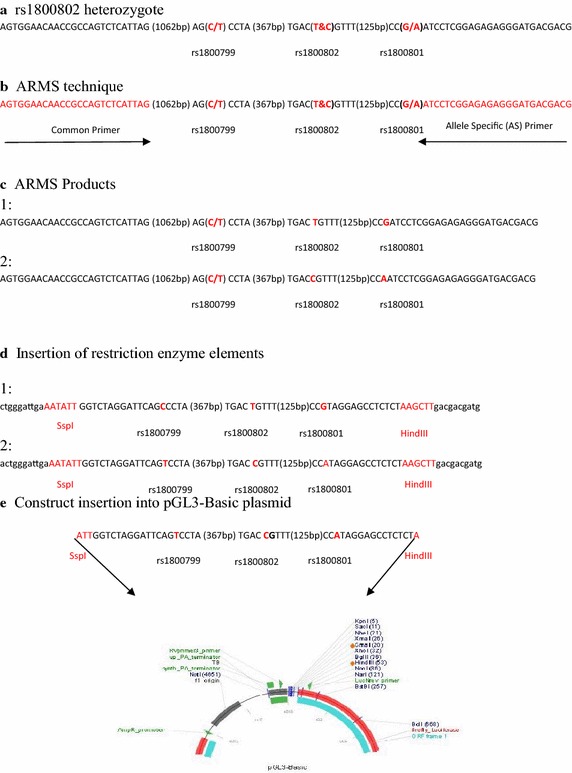
Construct preparation and plasmid insertion

The haplotype constructs (TCA and CTG) were obtained by direct haplotyping with ARMS and RFLP–PCR methods as reported in the previous study (Najafi et al. 2014). At first, the amplified two-allele fragments containing the rs1800802 heterozygote were separated by ARMS–PCR technique at the rs1800801 position. The ARMS–PCR reactions were performed in two microtubes (25 µl) containing MgCl_2_ (1.5 mM), Hot-start Taq plus DNA polymerase (1.25 U), genomic DNA (0.2 µg), Common Primer (1 µM; 5′-AGTGGAACAACCGCCAGTCTCATTAG-3′) and Allele Specific (AS) Primers (1 µM; 5′-GCAGCAGTAGGGAGAGAGGCTCCTAC-3′ and 5′-GCAGCAGTAGGGAGAGAGGCTCCTAT-3′). The temperature cycles (n = 30) were followed after incubation at 95 °C for 5 min (95 °C for 30 s, 68 °C for 50 s and 72 °C for 90 s) and a final extension at 72 °C for 7 min. Then, each allele was amplified with the designed forward and reverse primers to insert the digestible elements within constructs based on the previous PCR condition (Fig. 1).

### DNA cloning

#### Digestion and ligation

**Fig. 2:**
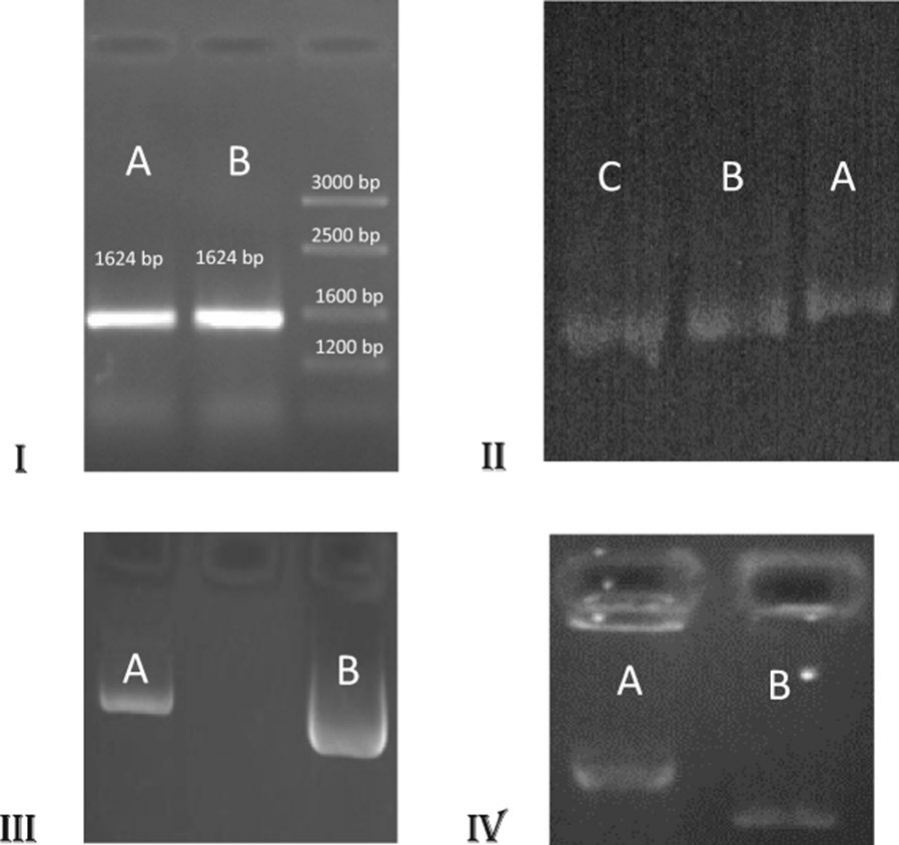
pGL3 Basic plasmid and constructs on agarose gel. **I** ARMS products; the ARMS–PCR reactions were performed in two microtubes and the products (1624 bp) were run on the agarose gel (2 %) electrophoresis (*Lanes A*, *B* and Ladder). **II** Constructs containing three polymorphic sites were digested using restriction enzymes (REs) and were run on the agarose gel (3.5 %) electrophoresis (*A* amplified product, 568 bp, *B* digestion with SspI, 555 bp, *C* digestion with SspI and HindIII, 540 bp). **III** pGL3 Basic plasmid was digested using restriction enzymes (SmaI and HindIII) and was run on the agarose gel (2 %) electrophoresis (*A* intact plasmid, *B *digested plasmid with REs). **IV** The REs-digested constructs (539 bp) were inserted and ligated into pGL3 Basic plasmid and were compared on the agarose gel (2 %) (*A* construct-inserted plasmid, *B* intact plasmid)

 The haplotype constructs were digested with SspI (NEB; Cat. No. R0132L, 10 U, overnight) and HindIII (NEB; Cat. No. R0104S, 8 U, overnight) restriction enzymes. Then, the fragments were evaluated on agarose gel 3.5 % (w/v). The pGL3-Basic plasmid (Promega; AC U47295) was also digested by SmaI (NEB; Cat. No. RO141L, 10 U, overnight) and HindIII (8 U, overnight) enzymes (Fig. 2).

Rapid DNA dephose and ligation kit (Roche; Cat. No. 11 635 379 001) was used for the ligation of fragments at the upstream region of reporter gene. The results were confirmed on agarose gel 2 % (w/v). Briefly, 1 µl (200–300 ng) of pGL3-Basic plasmid, 1 µl of fragment (20–50 ng), 10 µl of T4 DNA ligation buffer and 1.5 µl of T4 DNA ligase (7.5 U) were gently mixed and incubated for 30 min at room temperature followed up to 24 h at 16 °C (Figs. 1, 2).

### Cell culture

The human G292 osteoblast cells were harvested in Dulbecco’s modified Eagle’s medium (DMEM) supplemented with 5 % (v/v) fetal bovine serum (FBS) at 37 °C in a humidified CO2 atmosphere 5 %. The plasmid transfection was conducted by X-tremeGENE HP DNA transfection reagent (Roche; Cat. No. 06366244001) to introduce plasmids into cells essentially. They were transferred into 6-well plates (confluency; 70 %) and, were exposed for 4 h with 200 µl of transfection complex (2 µg of constructed plasmid and 6 µl X-tremeGENE). Then, the transfected cells (2 millions) were treated with phorbol 12-myristate 13-acetate (Sigma; Cat. No. P8139) for 2 h and, were grown for 24 h.

### RNA extraction and cDNA synthesis

Total RNA was isolated from the plasmid-transfected cells using HP RNA isolation kit (Roche; Cat. No. 11 828 665 001) as described by the manufacturer’s instructions. Genomic DNA was eliminated by DNase I during the extraction procedure. Briefly, the cells were washed three times using cold phosphate-buffered saline solution (PBS) and, were harvested in 400 µl of lysis-binding buffer followed by centrifugation to remove cellular debris. Then, wash buffer (400 µl) was added and centrifuged. After adding 50 µl of elution buffer, RNA was stored at −80 °C.

cDNA synthesis was carried out with QuantiTect Reverse Transcription kit (Qiagen; Cat. No. 205310) using random primers in a total volume of 20 µl. Quality of cDNA was tested through PCR reaction.

### Real-time qPCR technique

The luciferase expression level was measured with QuantiFast SYBER Green PCR kit (Qiagen; Cat. No. 204054) using specific primers (0.5 µM each). The reporter gene was located in the downstream of pGL3-Basic plasmid-inserted haplotype constructs and was not able to express in the cell model. The primers were designed by Genamics Expression software. Furthermore, the beta-actin (ACTB) gene was applied as endogenous gene (Table 1).

**Table 1:** Primers used for RT-qPCR reaction

Primer	Sequence	T_annealing_
F-luciferase	5′-CATAGCTTCTGCCAACCGAACG-3′	
R-luciferase	5′-GGAAGATGGAACCGCTGGAGAG-3′	68
F-ACTB	5′-GCGAGAAGATGACCCAGATCATG-3′	
R-ACTB	5′-CGTCACCGGAGTCCATCACG-3′	67

The temperature cycles (n = 40) for luciferase gene were followed after incubation at 95 °C for 5 min (95 °C for 10 s, 68 °C for 15 s and 72 °C for 15 s). Also, the temperature cycles (n = 40) for ACTB gene were followed after incubation at 95 °C for 5 min (95 °C for 15 s, 67 °C for 15 s and 72 °C for 15 s). Standard curves were composed of tenfold dilutions and, melting curves ranged from 65 to 95 °C by steps of 0.5 °C were performed to evaluate non-specific products.

### Statistical analysis

Statistical analysis was performed using statistical software package (SPSS 18, Chicago). Relative expression was measured and tested according to Pfaffl formula ($2^{{ -\Delta CT }}$). The expression levels from CTG (n = 6 wells) and from TCA (n = 6 wells) cell cultures were compared using Mann–Whitney U test. p Value <0.05 was considered to be significant.

## Results

**Fig. 3:**
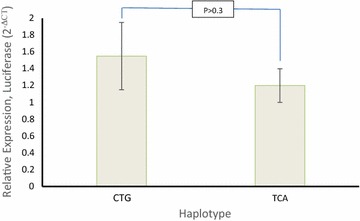
Relative expression level between high and low frequency haplotypes

TCA and CTG haplotypes (rs1800799, rs1800802 and rs1800801) were successfully constructed by the PCR techniques. The construct and insertion results were correctly considered on the agarose gel. The pGL3-Basic construct-inserted plasmids were transferred into osteoblast cells and, were subjected for reporter gene expression. The results showed that the relative expression of reporter gene (CTG 1.5 ± 0.4; TCA 1.2 ± 0.2) was not significantly different (p > 0.3). The findings showed that CTG and TCA haplotypes cannot functionality affect the expression patterns in human G292 osteoblast cells (Fig. 3).

## Discussion

Matrix Gla protein (84 aa, 14 kDa) is a secretary protein containing five γ-carboxyglutamic acid (Gla) residues originally isolated from bone (Schurgers et al. 2013). Its expression is also reported in other tissues including kidney, lung, heart and cartilage. Studies have also been reported the MGP gene expression in vascular smooth muscle cells (VSMCs), endothelial cells (ECs) and fibroblasts (Price et al. 1976).

In vitro studies revealed that the rs1800801 A variant had an approximately 1.5-fold higher activity than G variant in VSMCs (Farzaneh-Far et al. 2001). Herrmann et al. carried out transient transfection experiments with allele promoter–reporter gene constructs and DNA–protein interaction assays to investigate the functionality of promoter rs1800802 and rs1800801 variants. The minor rs1800802 C allele consistently conferred a reduced promoter activity of 20 % in rat vascular smooth muscle cells and of 50 % in a human fibroblast cell line whereas the other polymorphisms including rs1800801 displayed no evidence of in vitro functionality (Herrmann et al. 2000). The relationships were inconsistent and limited to independent roles of polymorphic sites. It was necessary to show the additive effects on transcription factor elements if there is any association between the mentioned polymorphic sites. In agreement with others (Farzaneh-Far et al. 2001; Crosier et al. 2009) and our previous report (Najafi et al. 2014), the population studies showed no difference between MGP concentration and promoter polymorphic variants. We also didn’t find the association between serum MGP concentrations and two-allele haplotypes in population using direct haplotyping and prediction tools. We confined our study to dominant and recessive haplotypes to show whether there is any association between polymorphic sites and also their phenotypes using transient transfection experiments. Other polymorphic sites present in promoter region might affect the gene expression patterns, potentially present also in our constructs, which have not the same genetic background of the haplotypes found in the population. However, these variants are present also in the control constructs. We aimed in fact to verify the differences in expression patterns due to the three SNPs, to test whether they are functional and the responsible of the phenotypic variability observed in the population. Although the dominant haplotype (CTG) showed slightly a higher expression than the recessive haplotype (TCA), the difference was not statistically significant. These results supported the population and prediction studies that the allele changes within the elements could not significantly affect the functionality of the promoter.

## Conclusions

We concluded that the high and low frequent polymorphic haplotypes within MGP promoter region have not the effect on functionality of promoter and, could not significantly affect the phenotype.
